# Totally implantable venous catheters for chemotherapy: experience in 500 patients

**DOI:** 10.1590/S1516-31802004000400003

**Published:** 2004-07-01

**Authors:** Nelson Wolosker, Guilherme Yazbek, Kenji Nishinari, Luiz Caetano Malavolta, Marco Antonio Munia, Marcel Langer, Antonio Eduardo Zerati

**Keywords:** Infection, Thrombosis, Catheterization, Central venous catheterization, Drug therapy, Infecção, Trombose, Cateterismo venoso central, Cateterismo, Quimioterapia

## Abstract

**CONTEXT:**

Totally implantable devices are increasingly being utilized for chemotherapy treatment of oncological patients, although few studies have been done in our environment to analyze the results obtained from the implantation and utilization of such catheters.

**OBJECTIVE:**

To study the results obtained from the implantation of totally implantable catheters in patients submitted to chemotherapy.

**TYPE OF STUDY:**

Prospective.

**SETTING:**

Hospital do Câncer A.C. Camargo, São Paulo, Brazil.

**METHODS:**

519 totally implantable catheters were placed in 500 patients submitted to chemotherapy, with preference for the use of the right external jugular vein. Evaluations were made of the early and late-stage complications and patient evolution until removal of the device, death or the end of the treatment.

**RESULTS:**

The prospective analysis showed an average duration of 353 days for the catheters. There were 427 (82.2%) catheters with no complications. Among the early complications observed, there were 15 pathway hematomas, 8 cases of thrombophlebitis of the distal stump of the external jugular vein and one case of pocket infection. Among the late-stage complications observed, there were 43 infectious complications (0.23/1000 days of catheter use), 11 obstructions (0.06/1000 days of catheter use) and 14 cases of deep vein thrombosis (0.07/1000 days of catheter use). Removal of 101 catheters was performed: 35 due to complications and 66 upon terminating the treatment. A total of 240 patients died while the catheter was functioning and 178 patients are still making use of the catheter.

**CONCLUSION:**

The low rate of complications obtained in this study confirms the safety and convenience of the use of totally implantable accesses in patients undergoing prolonged chemotherapy regimes.

## RESUMO

**CONTEXTO:**

Os dispositivos totalmente implantáveis vêm sendo cada vez mais utilizados para quimioterapia de pacientes oncológicos, porém poucos são os estudos em nosso meio que analisam os resultados obtidos com a implantação e utilização desses cateteres.

**OBJETIVO:**

Estudar os resultados obtidos com a implantação de cateteres totalmente implantáveis em pacientes submetidos a quimioterapia.

**TIPO DO ESTUDO:**

Prospectivo.

**LOCAL:**

Hospital do Câncer A.C. Camargo, São Paulo, Brasil.

**MÉTODOS:**

Foram colocados 519 cateteres totalmente implantáveis em 500 pacientes a serem submetidos a regime de quimioterapia preferencialmente utilizando-se a veia jugular externa direita. Foram avaliadas as complicações precoces, as tardias e a evolução até a retirada do dispositivo, morte ou fim de tratamento.

**RESULTADOS:**

A análise prospectiva mostrou duração média dos cateteres de 353 dias. Em 427 (82,2%) cateteres não se observou nenhuma complicação. Entre as complicações precoces, observamos 15 hematomas de trajeto, oito tromboflebites de coto distal de veia jugular externa e uma infecção de bolsa de subcutâneo. Entre as complicações tardias, observamos 43 complicações infecciosas (0,23/1000 dias de uso de cateter), 11 obstruções (0,06/1000 dias de uso de cateter) e 14 tromboses venosas profundas (0,07/1000 dias de uso de cateter). Foram retirados 101 cateteres, 35 devido às complicações e 66 por final de tratamento. 240 pacientes foram a óbito com o cateter funcionante e 178 pacientes ainda o utilizavam para quimioterapia.

**CONCLUSÃO:**

As baixas taxas de complicação obtidas nesse estudo confirmam a segurança e conveniência do uso dos acessos totalmente implantáveis em pacientes em regime prolongado de quimioterapia.

## INTRODUCTION

Vascular access has great importance in the treatment of patients submitted to prolonged endovenous therapy. Since the introduction of the partially implantable catheter described by Broviac^1^ and modified by Hickman^2^ during the 1970s, handling oncological patients has become much easier due to the increased safety in relation to the previously performed peripheral or temporary vein accesses. With the introduction of totally implantable catheters in the 1980s, the choices have become even greater, thus revolutionizing the treatment of cancer patients.

Totally implantable catheters consist of silicone catheters whose distal extremity is positioned at the junction of the superior vena cava with the right atrium and whose proximal extremity is connected to a pocket inserted into subcutaneous tissue, usually in the anterior wall of the thorax. This allows safe access to the vascular system for endovenous treatments.^3,4^

Because this equipment is totally implantable, without any of its components brought to the surface through the skin, it offers advantages over partially implantable systems:^5^ low infection rates and no restrictions on patients’ physical activities.^2^ These advantages have been leading to increasingly frequent use of such systems, especially for outpatient chemotherapy treatment of neoplasms and acquired immunodeficiency (aids).

Although totally implantable catheters have become widespread over recent years, few studies have yet been done in our country, especially prospective studies with significant numbers of cases and long-term follow-up.

## OBJECTIVE

The goal of this work was to prospectively study the results obtained from the implantation of 519 totally implantable catheters in 500 patients, utilized for an average duration of 353 days, in patients submitted to chemo-therapy in a large-sized hospital with its own dedicated vascular clinical team.

## PATIENTS AND METHODS

From September 1997 to September 2002, 519 totally implantable catheters were inserted into 500 cancer patients for chemo-therapy. Their ages ranged from 14 to 79 years, with an average of 50.5 years; 376 were female (75.2%) and 124 were male (24.8%).

The main clinical indications for the implantation of the catheters were chemotherapy for the treatment of solid tumors (85.2%) and hematological diseases (14.8%). The diagnoses of the tumors that led to catheter indication are to be found in Table 1.

**Table 1:** Diagnoses of the tumors that led to the indication of chemotherapy for 500 patients in a cancer treatment service

Tumor	n
Breast	226 (45.2%)
Lymphoma, leukemia	74 (14.8%)
Digestive tract	48 (9.6%)
Ovaries, uterus, prostate, bladder, testes	37 (7.4%)
Urological	32 (6.4%)
Soft tissues	18 (3.6%)
Skin	18 (3.6%)
Lung	12 (2.4%)
Others	35 (7.0%)
**Total**	**500 (100%)**

*n = number of patients.*

All the implantation procedures were performed in the operating room, with the participation of an anesthetist monitoring the operation, independent of the type of anesthesia utilized. General anesthesia was used in 233 cases (44.9%), local anesthesia in association with endovenous sedation in 202 cases (38.9%) and local anesthesia alone in 84 cases (16.2%).

The totally implantable catheters utilized consisted of 9.6-F silicone catheters and titanium or silicone pockets. Because of their structural characteristics and the manner of accessing them (percutaneous puncture of the pocket), they only allow restricted flow and reflux, thereby making it possible to inject medications but not to perform blood collection and transfusion.

No catheter was introduced while there was the presence of fever of indeterminate origin, any systemic infectious condition (bacteremia or septicemia), or signs of skin infection, in the proximity of the location for implanting the pocket.

The preferred access route was the external jugular vein. The techniques employed were dissection or puncture, depending on the local anatomical conditions for catheter implantation. Table 2 presents the access routes and techniques employed.

**Table 2:** Access routes and insertion techniques utilized for catheter placement in 500 patients receiving chemotherapy in a cancer treatment service

Vein access route	Dissection	Puncture	Total
Right external jugular	264	0	264 (50.8%)
Left external jugular	119	0	119 (22.9%)
Right internal jugular	9	48	57 (10.9%)
Left internal jugular	17	25	42 (8.1%)
Right cephalic	6	0	6 (1.2%)
Left cephalic	1	0	1 (0.2%)
Right subclavian	0	5	5 (1.0%)
Left subclavian	0	5	5 (1.0%)
Right common femoral	2	4	6 (1.2%)
Left common femoral	0	9	9(1.7%)
Right saphenous	2	0	2 (0.4%)
Left saphenous	3	0	3 (0.6%)
**TOTAL**	**423**	**96**	**519 (100%)**

Cardiac arrhythmia was observed after insertion of the guide wire in 13 of the 93 procedures performed using the puncture technique. These cases evolved satisfactorily through simply pulling the guide wire as far as the superior vena cava. Because of the satisfactory evolution in such cases, these were considered not as complications, but as frequent events.

In 39 cases, it was necessary to alter the access route initially proposed. In 26 cases, this occurred because it was impossible to advance the catheter via the external jugular vein due to incompatibility of its diameters with the catheter. In 13 cases, this occurred because the catheter did not advance to the central position and correct positioning was not achievable via the initial access route.

All the catheters were implanted under fluoroscopic control (Philips BV 300) for correct positioning of the extremity at the entrance to the right atrium.^6-8^ In addition to this, the flow and reflux were always tested via the pocket. When these were unsatisfactory, a situation that was recognized during the operation, the catheter extremity was repositioned until adequate functioning was obtained. After terminating the procedure, occlusive dressings were placed on the incisions. The device was filled with a heparin solution diluted with physiological serum to a concentration of 50 U/ml. The procedure duration varied from 20 to 135 minutes, with an average of 52 minutes.

During the patient's internment, dressing changes were performed by the nursing team.^9^ Phlogistic signs were sought from the pocket, subcutaneous tunnel and incisions. The nursing team advised the patients and their relatives to remain vigilant and keep changing the dressings during the homestay period.

The totally implantable catheters had the function of allowing the infusion of drugs for chemotherapy. Once implanted, the system was immediately ready for use. At the end of treatment, the catheters were removed because of the inherent risk of complications like infections and venous thrombosis. This procedure was always performed in the operating room, on an elective basis, with the use of local anesthesia in association with sedation.

The patients were prospectively followed up until the time of catheter removal or death. The length of utilization of these catheters and their early and late-stage complications, and also the treatment utilized for correcting such complications, were studied.

We considered early complications to be those that occurred between the operation and the completion of two weeks of follow-up. The late-stage complications were those that occurred after this period of initial evaluation.

## RESULTS

Of the 519 catheters implanted, 92 (17.7%) presented some type of complication. Removal of 101 catheters was performed, of which 66 (65.4%) were elective indications due to the termination of the treatment and 35 (34.6%) resulted from complications that could not be controlled using clinical measures. In 240 patients (48%), the catheter remained functional until the patient's death, and 178 patients (35.6%) are still making use of their catheters for clinical treatment. The total catheter use was 183,467 days, ranging from 5 to 1,729 days per patient, with an average of 353 days.

The early complications observed were 15 pathway hematomas that were successfully treated by clinical means, eight patients with thrombophlebitis of the distal stump of the external jugular vein, who also evolved satisfactorily with clinical treatment, and one case of subcutaneous pocket infection with systemic infectious manifestations, in which the totally implantable catheter was removed 11 days after its introduction. We did not have any cases of pneumothorax, arterial lesion or catheter emplacement failure.

The other complications observed occurred at a later stage and involved 68 of the 519 catheters implanted (13.1%). In three catheters, more than one complication per catheter was observed during the follow-up: one of these patients had infections and venous thrombosis, and two had infections and occlusion.

Infectious complications occurred in relation to 43 of the catheters (8.2%), at a frequency of 0.23/1,000 days of catheter use. Non-complicated primary bacteremia (fever and shivers only) occurred in 27 cases and were treated using peripheral endovenous antibiotic therapy (vancomycin) for the first 48 hours, followed by infusion of this drug via the catheter. The catheter had to be removed in 9 cases because the fever continued or the patient's clinical state worsened. In 18 cases there was an improvement in the clinical condition, with the catheters preserved (preservation rate of 66.6%). Subcutaneous pocket infection occurred in 16 patients and all of them were treated with endovenous antibiotic therapy and catheter removal.

Non-infectious complications occurred in 28 cases. In 11 of them, there was obstruction of the catheter, representing an incidence of 0.06 obstructions per 1,000 days of catheter use. In 14 cases, there was deep vein thrombosis associated with the catheter, representing 0.07 cases per 1,000 days of catheter use. In the cases of obstruction, the treatment employed was local fibrinolysis with the use of streptokinase solution at a concentration of 12,500 U/ml. The solution was maintained for one hour and then a catheter function test was performed. The local fibrinolysis was repeated every hour up to the fourth attempt. In 8 cases, normal catheter function was reestablished (preservation rate of 72.7%). In three cases, unblocking of the obstruction was impossible and the catheter had to be removed, with subsequent scheduling of a new access.

We observed 14 cases of deep vein thrombosis: six affecting the subclavian veins, six the internal jugular veins, one the superior vena cava and one the iliac vein. By implementing systemic anticoagulation using heparin of low molecular weight and warfarin, it was possible to preserve 11 of these 14 catheters (preservation rate of 78.5%). The catheters were removed only in the three cases of deep vein thrombosis that were associated with non-functioning catheters. Neither of these cases progressed with pulmonary thromboembolism.

One case of pocket extrusion, one extravasation of medication related to rupture of the totally implantable catheter and one tip migration were observed. In all these cases the totally implantable catheters were removed.

## DISCUSSION

Since the introduction of totally implantable catheters, the treatment of oncological patients has become much better because such venous accesses allow the safe infusion of chemotherapy over long periods.^10-12^ In addition to this, such devices foster improved quality of life for patients, with an absence of restrictions on their activities.^2^

The increased indication of these devices reflects their acceptance in the day-by-day routine of oncology centers. One small disadvantage is the cost of the procedure and of the device itself. However, despite the great usefulness of these catheters, their insertion and maintenance is not free from complications.

The preferred access route for implanting most of the long-duration catheters in our service is via dissection of the external jugular vein, which was successfully utilized in 73.7% of our cases. This vein provides a safe route because it is a superficial vein. Its dissection requires minimal tissue manipulation and allows easy control of possible complications. Most totally implantable (9.6 F) and semi-im-plantable (12 F) catheters can be inserted via this route, which is usually only unsuitable for apheresis catheters due to caliber incompatibility (13.5 F) and the frequent kinking of the catheter at the entrance to the jugular vein.

Although dissection of the external jugular vein has been little used in other services, in which the preferred route is via puncture of the subclavian vein,^10^ our route has proven to be suitable and also safer in relation to complications, considering that we did not have any cases of pneumothorax, hemothorax, arterial lesion or pinching of the catheter between the first costal arch and the clavicle.

The experience of the surgical team is essential to the success of the procedure. The surgeon needs to be accustomed to dealing with different access routes, in order to offer the safest route, as well as being prepared for possible changes in intraoperative conduct, which in our series occurred in 7.5% of the procedures.

General anesthesia was the most used in our cases (44.9%) because it provides greater comfort for patients and reassurance for the surgical team when dealing with the possible complications inherent in the operation.

We did not utilize prophylactic antibiotic therapy because there is no clear evidence demonstrating decrease in the infectious rates through its use.^13,14^ In our series, the rate of early infection (related to the procedure and potentially treatable using prophylactic antibiotic therapy) was 0.19%.

With the improvement in the implantation techniques and equipment, complication rates have been diminishing. However, recent series in the literature show significant complication rates, with up to 2% incidence of pneumothorax, 14% cardiac arrhythmia, 3% arterial puncture, 3% bending of the guide wire, 3% kinking of the introductory sheath and 1% serious bleeding.^6,10,15^ In our series, because we preferentially utilized dissection of the jugular veins (81.5% of the total), we did not observe such complications. Even in the cases in which puncturing was performed (18.4% of the total), the incidence of pneumothorax and serious bleeding was null. We only observed cardiac arrhythmia caused by the introduction of the guide wire in 13 cases, which regressed in all these cases when the wire was pulled. The small number of complications during the operations can be attributed to the preoperative care (pre-anesthesia evaluation), the utilization of radioscopy equipment, standardized surgical techniques and the specialized team.

The non-infectious early complications were 15 hematomas in the subcutaneous tunnel, in thrombocytopenic patients (despite the pre and intraoperative transfusion of platelets), and eight cases of phlebitis of the distal stump of the external jugular vein, which presented good evolution. On the other hand, in one case of early infection of the subcutaneous pocket, there was the appearance of clinical manifestations and early removal of the catheter was necessary.

Late complications are of fundamental importance, since these are the main causes of catheter removal,^15^ and they occurred in 68 of our implanted catheters. Despite the care, infection continues to be the main late complication, with bacteremia related to the catheter being the most frequent type. In the different series published, the infection rate has reached up to 31%. In our sample, we observed such complications in 8.2% of the patients, a rate of 0.23 per 1,000 days of use, which is comparable with the literature (Table 3).

We can subdivide the infections into subcutaneous pocket infection and bacteremia related to manipulation of the catheter.^16,17^ The diagnosis of infections is routinely based on the clinical condition. When cutaneous hyperemia occurs in the catheter pathway (subcutaneous pocket) or the incisions, the diagnosis is clear. Nonetheless, in 62.7% of the cases we only observed fever without a determinate focus. It is difficult to obtain diagnostic proof through laboratory tests (cultures) in bacteremia cases because of the low sensitivity of such tests,^18-23^ and for this reason the clinical condition must be utilized for indicating the need for catheter removal. Even cultures from the catheter present low sensitivity and thus must not be used as definitive diagnostic criteria. Monitoring of the clinical improvement is the best parameter.

In many cases of bacteremia related to the catheter, antibiotic therapy without catheter removal is an option since the patient is stable and without signs of sepsis. When there is diagnostic suspicion of infection (fever without apparent focus or catheter manipulation), peripheral blood and catheter cultures are performed. Immediately after this, antibiotic therapy is begun for a period of 48 hours via a peripheral vein, followed by infusion via the catheter for a period of 14 to 21 days. The antibiotic we utilized empirically was vancomycin, because of the high incidence of infection by coagulase-negative Staphylococcus.^22^ In the literature, the catheter preservation rate is between 60 and 80%,^16^ while in our sample we obtained therapeutic success in 75% of the cases.

We can divide the most important non-infectious complications into obstructions of the distal extremity of the catheter and deep vein thrombosis. Their incidence, too, is far from negligible. In the literature the rates range from 7 to 50%.^24-27^ In our series we observed 25 complications (4.8%), with 0.06 obstructions and 0.07 deep vein thromboses per 1,000 days of catheter use, which is comparable with the literature (Table 3). Some factors are associated with a greater tendency towards deep vein thrombosis among patients submitted to chemotherapy, such as endothelial lesions (related to the catheter) and reduction in proteins C and S caused by the chemotherapy regime.^28-30^ The prophylactic use of low doses of oral anticoagulant for diminishing the incidence of such complications is discussed in the literature.^10^

**Table 3:** Rates of complications after catheter implantation in the literature and our data

	Number of catheters	Days in *situ*	Infection	Deep vein	Obstruction thrombosis
Bothe et al. 1984^32^	75	90	1.0/1000	0.6/1000	-
Lokich et al. 1985^33^	92	127	0.6/1000	1.2/1000	-
Harvey et al. 1989^34^	198	330	0.4/1000	0.3/1000	-
Freytes et al. 1990^4^	134	305	0.02/1000	0.03/1000	0.04/1000
Torramadé et al. 1993^35^	234	277	0.2/1000	0.1/1000	0.2/1000
Biffi et al. 1997^36^	178	180	0.16/1000	0.062/1000	-
Our data	519	353	0.23/1000	0.07/1000	0.06/1000

In our sample, we encountered 14 cases of deep vein thrombosis (2.7%), but catheter removal was only necessary for three patients (0.57%), due to non-functioning of the catheter. We did not observe any cases of pulmonary thromboembolism, and for this reason we think that totally implantable catheters do not have to be removed indiscriminately, because of the inherent risk in such treatment, unless there is associated catheter obstruction.

The clinical suspicions of deep vein thrombosis were based on the presence of edema, pain, erythrocyanosis and collateral circulation in the limb. Diagnostic confirmation of deep vein thrombosis was obtained by duplex scanning. The treatment employed was conservative when the catheter was functioning. Systemic anticoagulation was performed, initially using heparin of low molecular weight and subsequently using warfarin, thus avoiding systemic fibrinolysis due to the inherent risks of bleeding in such patients.^31^

The cases of obstruction of the distal end of the catheter presented good evolution. The local fibrinolysis utilized in these cases was effective in eight of the 11 patients (preservation rate of 72.7%).

## CONCLUSION

The low rate of complications implying catheter loss in this study confirms the safety and convenience of the use of totally implantable accesses in patients undergoing prolonged chemotherapy regimes.

Systematization of the operative technique, as well as the maintenance of a specialized multidisciplinary team contributes towards the reduction of early complications, thereby making the operation safe. Guidance and follow-up for patients over the course of the treatment prevents the late complications.

## References

[1] Broviac JW, Cole JJ, Scribner BH (1973). A silicone rubber atrial catheter for prolonged parenteral alimentation. Surg Gynecol Obstet.

[2] Hickman RO, Buckner CD, Clift RA, Sandres JE, Stewart P, Thomas ED (1979). A modified right atrial catheter for access to the venous system in marrow transplant recipients. Surg Gynecol Obstet.

[3] Gyves JW, Ensminger WD, Niederhuber JE (1984). A totally implanted injection port system for blood sampling and chemotherapy administration. JAMA.

[4] Freytes CO, Reid P, Smith KL (1990). Long-term experience with a totally implanted catheter system in cancer patients. J Surg Oncol.

[5] Carde P, Cosset-Delaigue MF, Laplanche A, Chareau I (1989). Classical external indwelling central venous catheter versus totally implanted venous access systems for chemotherapy administration: a randomized trial in 100 patients with solid tumors. Eur J Cancer Clin Oncol.

[6] Capaccioli L, Nistri M, Distante V, Rontini M, Manetti A, Stecco A (1998). [Insertion and management of long-term central venous devices: role of radiologic imaging techniques]. Posizionamento e gestione dei sistemi per accesso venoso centrale a lungo termine: contributo della diagnostica per immagini. Radiol Med (Torino).

[7] Cohn DE, Mutch DG, Rader JS, Farrell M, Awantang R, Herzog TJ (2001). Factors predicting subcutaneous implanted central venous port function: the relationship between catheter tip location and port failure in patients with gynecologic malignancies. Gynecol Oncol.

[8] Herrmann KA, Waggershauser T, Helmberger T, Heinemann V, Sittek H, Reiser M (1999). [Percutaneous interventional radiologic implantation of intravenous port-catheter systems]. Interventionell-radiologische perkutane Implantation intravenöser Port-Katheter-Systeme. Radiologe.

[9] Campbell T (2000). Central venous catheter management in patients with cancer. Int J Palliat Nurs.

[10] Minassian VA, Sood AK, Lowe P, Sorosky JI, Al-Jurf AS, Buller RE (2000). Longterm central venous access in gynecologic cancer patients. J Am Coll Surg.

[11] Abrahm JL, Mullen JL (1982). A prospective study of prolonged central venous access in leukemia. JAMA.

[12] Costantini R, Napolitano L, Scurti D, Innocenti P (1997). [Totally implantable central venous systems in neoplastic patients]. I sistemi venosi centrali totalmente impiantabili nei pazienti neoplastici. Ann Ital Chir.

[13] Jaeger K, Osthaus A, Heine J (2001). Efficacy of a benzalkonium chloride-impregnated central venous catheter to prevent catheter-associated infection in cancer patients. Chemotherapy.

[14] Ranson MR, Oppenheim BA, Jackson A, Kamthan AG, Scarffe JH (1990). Double-blind placebo controlled study of vancomycin prophylaxis for central venous catheter insertion in cancer patients. J Hosp Infect.

[15] Ballarini C, Intra M, Pisani Ceretti A (1999). Complications of subcutaneous infusion port in the general oncology population. Oncology.

[16] Sotir MJ, Lewis C, Bisher EW, Ray SM, Soucie JM, Blumberg HM (1999). Epidemiology of device-associated infections related to a long-term implantable vascular access device. Infect Control Hosp Epidemiol.

[17] Douard MC, Ardoin C, Payri L, Tarot JP (1999). [Infectious complications of long term intravenous devices: incidence, risk factors, diagnostic tools]. Complications infectieuses des dispositifs intraveineux de longue durée: incidence, facteurs de risque, moyens diagnostiques. Pathol Biol (Paris).

[18] Blot F, Nitenberg G, Chachaty E (1999). Diagnosis of catheter-related bacteraemia: a prospective comparison of the time to positivity of hub-blood versus peripheral-blood cultures. Lancet.

[19] Groebli Y, Bartolomei J, Wuthrich P, Callewaert G, Tschantz P, Piguet D (1998). [Post-mortem evaluation following oncologic treatment of the functional and bacteriologic status of 25 permanent venous access devices]. Evaluation post-mortem après traitement oncologique de l’état fonctionnel et bactériologique de 25 accès veineux permanents. Schweiz Med Wochenschr.

[20] Raad I, Costerton W, Sabharwal U, Sacilowski M, Anaissie E, Bodey GP (1993). Ultrastructural analysis of indwelling vascular catheters: a quantitative relationship between luminal colonization and duration of placement. J Infect Dis.

[21] Astagneau P, Maugat S, Tran-Minh T (1999). Long-term central venous catheter infection in HIV-infected and cancer patients: a multicenter cohort study. Infect Control Hosp Epidemiol.

[22] Kappers-Klunne MC, Degener JE, Stijnen T, Abels J (1989). Complications from long-term indwelling central venous catheters in hematologic patients with special reference to infection. Cancer.

[23] Hanna HA, Raad I (2001). Blood products: a significant risk factor for long-term catheter-related bloodstream infections in cancer patients. Infect Control Hosp Epidemiol.

[24] Balestreri L, De Cicco M, Matovic M, Coran F, Morassut S (1995). Central venous catheter-related thrombosis in clinically asymptomatic oncologic patients: a phlebographic study. Eur J Radiol.

[25] Gould JR, Carloss HW, Skinner WL (1993). Groshong catheter-associated subclavian venous thrombosis. Am J Med.

[26] Pintor Holguín E, Sáez Noguero F, Piret Ceballos MV (1997). [Axillary-subclavian thrombosis: review of its etiology and features in recent years]. Trombosis axilosubclavia: revisión de la etiología y características en los últimos años. An Med Interna.

[27] De Cicco M, Matovic M, Balestreri L (1997). Central venous thrombosis: an early and frequent complication in cancer patients bearing long-term silastic catheter. A prospective study. Thromb Res.

[28] Conlan MG, Haire WD, Lieberman RP, Lund G, Kessinger A, Armitage JO (1991). Catheter-related thrombosis in patients with refractory lymphoma undergoing autologous stem cell transplantation. Bone Marrow Transplant.

[29] Monreal M, Davant E (2001). Thrombotic complications of central venous catheters in cancer patients. Acta Haematol.

[30] Raad II, Luna M, Khalil SA, Costerton JW, Lam C, Bodey GP (1994). The relationship between the thrombotic and infectious complications of central venous catheters. JAMA.

[31] Rodenhuis S, van't Hek LG, Vlasveld LT, Kroger R, Dubbelman R, van Tol RG (1993). Central venous catheter associated thrombosis of major veins: thrombolytic treatment with recombinant tissue plasminogen activator. Thorax.

[32] Bothe A, Piccione W, Ambrosino JJ, Benotti PN, Lokich JJ (1984). Implantable central venous access system. Am J Surg.

[33] Lokich JJ, Bothe A, Benotti P, Moore C (1985). Complications and management of implanted venous access catheters. J Clin Oncol.

[34] Harvey WH, Pick TE, Reed K, Solenberger RI (1989). A prospective evaluation of the Port-A-Cath implantable venous access system in chronically ill adults and children. Surg Gynecol Obstet.

[35] Torramadé JR, Cienfuegos JA, Hernández JL (1993). The complications of central venous access systems: a study of 218 patients. Eur J Surg.

[36] Biffi R, de Braud F, Orsi F, Pozzi S, Mauri S, Goldhirsch A, Nolè F, Andreoni B (1998). Totally implantable central venous access ports for long-term chemotherapy. A prospective study analyzing complications and costs of 333 devices with a minimum follow-up of 180 days. Ann Oncol.

